# An RNAi based screen in *Drosophila* larvae identifies fascin as a regulator of myoblast fusion and myotendinous junction structure

**DOI:** 10.1186/s13395-018-0159-9

**Published:** 2018-04-06

**Authors:** Jaclyn M. Camuglia, Torrey R. Mandigo, Richard Moschella, Jenna Mark, Christine H. Hudson, Derek Sheen, Eric S. Folker

**Affiliations:** Biology Department, Boston College, Chestnut Hill, MA 02467 USA

**Keywords:** Nuclear movement, Drosophila, Myoblast fusion, Myotendinous junction, Fascin, Myogenesis

## Abstract

**Background:**

A strength of *Drosophila* as a model system is its utility as a tool to screen for novel regulators of various functional and developmental processes. However, the utility of *Drosophila* as a screening tool is dependent on the speed and simplicity of the assay used.

**Methods:**

Here, we use larval locomotion as an assay to identify novel regulators of skeletal muscle function. We combined this assay with muscle-specific depletion of 82 genes to identify genes that impact muscle function by their expression in muscle cells. The data from the screen were supported with characterization of the muscle pattern in embryos and larvae that had disrupted expression of the strongest hit from the screen.

**Results:**

With this assay, we showed that 12/82 tested genes regulate muscle function. Intriguingly, the disruption of five genes caused an increase in muscle function, illustrating that mechanisms that reduce muscle function exist and that the larval locomotion assay is sufficiently quantitative to identify conditions that both increase and decrease muscle function. We extended the data from this screen and tested the mechanism by which the strongest hit, fascin, impacted muscle function. Compared to controls, animals in which fascin expression was disrupted with either a mutant allele or muscle-specific expression of RNAi had fewer muscles, smaller muscles, muscles with fewer nuclei, and muscles with disrupted myotendinous junctions. However, expression of RNAi against fascin only after the muscle had finished embryonic development did not recapitulate any of these phenotypes.

**Conclusions:**

These data suggest that muscle function is reduced due to impaired myoblast fusion, muscle growth, and muscle attachment. Together, these data demonstrate the utility of *Drosophila* larval locomotion as an assay for the identification of novel regulators of muscle development and implicate *fascin* as necessary for embryonic muscle development.

**Electronic supplementary material:**

The online version of this article (10.1186/s13395-018-0159-9) contains supplementary material, which is available to authorized users.

## Background

Skeletal muscle has a distinctive architecture that is generated by a unique set of developmental phases. Making the myofiber syncytium requires the fusion of mononucleated myoblasts. In both *Drosophila* and mammalian systems, individual myoblasts invade growing myotubes and deposit their nucleus into the common cytoplasm to drive myotube growth [1, 2]. Myoblast fusion is an actin-dependent process that is reminiscent of a cancer cell invading a tissue during metastasis [3]. The mononucleated myoblast extends a protrusive invadapodia-like structure that makes possible the penetration of the myotube and the mixing of cytoplasm. Many factors and signaling pathways that regulate myoblast fusion have been identified [2, 4, 5]. However, many of the genes necessary for invadapodia-like structures in other contexts have not yet been implicated in myoblast fusion, suggesting that additional regulators remain to be identified. One glaring omission from the categories of proteins that have been identified as regulators of myoblast fusion is proteins that stabilize filopodia. Invadapodia are filopodia-like structures [6, 7], and they require several factors that are known to stabilize filopodia. Furthermore, although loss-of-function data is lacking, dominant negative mutants of the formin Diaphanous inhibit myoblast fusion and may suggest that filopodia are essential for myoblast fusion [8, 9]. Thus, it is likely that additional factors that have been identified as capable of stabilizing filopodia for the purpose of protrusion and invasion in other contexts contribute to myoblast fusion.

Beyond myoblast fusion, there are several features of muscle development that either require, or have been hypothesized to require, precise regulation of the actin cytoskeleton, including the positioning of nuclei and the development of the myotendinous junction (MTJ). To date, evidence for actin-dependent nuclear movement in muscle is restricted to the squeezing of nuclei to the periphery of the muscle [10, 11], although it has been proposed that actin may contribute to the movement of nuclei along the length of the muscle [12, 13]. The role of actin in MTJ development is more established. MTJs are integrin-based adhesions that transmit force from the muscle to the skeleton [14]. The initial formation involves extension of filopodia-like structures from the muscle cell that interact with the tendon cell before forming a stable and somewhat rigid attachment that enables effective force transmission [15, 16]. All of these processes require linear actin-cables. The similarity in the actin-based structures suggests that the same molecular components may contribute to each of these aspects of muscle development. Therefore, it is critical to determine how newly identified genes and proteins contribute to each process.

Because the developmental path and final architecture of muscle cells is conserved from *Drosophila* to humans, flies provide a genetically tractable and inexpensive model for the identification of genes that are necessary for muscle development. Indeed, many screens for regulators of muscle development have been completed. Researchers have used adult locomotion [17] and embryonic muscle structure [18] as indicators of muscle development. Although these strategies have proven effective, they each have drawbacks. Analysis of embryonic muscle structure is labor intensive and requires significant expertise in muscle biology. Analysis of adult locomotion is limited because the disruption of many genes is lethal during pupation. Therefore, we have developed a simple assay for muscle function based on the larval locomotion. We have used this assay to screen for novel regulators of muscle function and identified fascin as one such regulator. Subsequent cell biological analysis implicates *fascin* as a regulator of myoblast fusion and MTJ structure.

## Methods

### *Drosophila* genetics

All stocks were grown under standard conditions at 25 °C. The *Fascin*^*sn28*^ allele was a generous gift from Tina Tootle (University of Iowa). All UAS-RNAi *Drosophila* lines were purchased from Bloomington Drosophila Stock Center. UAS-RNAi constructs were driven specifically in the mesoderm using *twist-GAL4*, which drives expression in the mesoderm from stage 10 of embryonic development through stage 13 of embryonic development, *DMef2-GAL4* that drives expression in the muscles from stage 12 through adulthood, or *MHC-GAL4* which drives expression in muscle from stage 17 of embryonic development through adulthood.

### Larval locomotion assay

We performed a modified version of the previously used assay that has been used to measure larval locomotion in individual larvae [19]. Virgins expressing *DMef2-GAL4* were mixed with males that carried the UAS-RNAi for 1 h in a vial with standard *Drosophila* food. After 1 h, adults were moved to a new vial and the embryos laid during the 1 h period were aged for 5 days until they were third-instar larvae (L3). Larvae were then floated from the food by the addition of 15% sucrose. Using a paintbrush, larvae were moved to a plate with wet yeast. After all of the genotypes had been collected, 10 larvae of each RNAi were moved to an arena that consisted of 3% agarose dyed black with standard food color poured over the top of a 96-well plate cover. Movement of larvae toward a stick dipped in ethyl butyrate was captured using an iPhone mounted above the arena. The speed of each larva was then analyzed using ImageJ.

### Viability assay

Stage 16 embryos were picked and placed on an agarose plate. The selected embryos were incubated at 20 °C overnight. After incubation, the number of unhatched embryos were counted to determine embryonic lethality and the L1 larvae from the hatched embryos were transferred to a vial of standard fly food. Larvae were incubated at 25 °C until larvae began to pupate and eclose. Adult flies were counted to determine pupal lethality and removed from the vials of standard fly food upon eclosure. After 1 week of no eclosures, the number of pupal cases were counted to determine larval lethality.

### Immunohistochemistry

#### Preparation of embryos

Embryos were collected at 25 °C and were dechorionated by submersion in 50% bleach for 4 min. Embryos were then fixed in a solution of equal parts heptane and 10% formalin (Sigma, Product # HT501128). Fixation lasted for 20 min during which time the embryos were placed on an orbital shaker that rotated at a rate of 250/min. Following fixation, the formalin and heptane were removed and replaced with a solution of equal parts methanol and heptane. The embryos were vortexed for 1 min to devitellinize the embryos. Embryos were stored in methanol at − 20 °C until immunostaining.

#### Preparation of larvae

Dissection of larvae was carried out as previously described [18] with minor modifications. The primary difference being that the buffer used was modified to increase the preservation of muscle structure. The modified dissection buffer was 100 mM PIPES (Sigma-Aldrich, P6757), 115 mM d-sucrose (Fisher Scientific, BP220-1), 5 mM trehalose (Acros Organics, 182550250), 10 mM sodium bicarbonate (Fisher Scientific, BP328-500), 75 mM potassium chloride (Fisher Scientific, P333-500), 4 mM magnesium chloride (Sigma-Aldrich, M1028), and 1 mM EGTA (Fisher Scientific, 28-071-G). Larvae were then fixed with 10% formalin (Sigma-Aldrich, HT501128) for 20 min. Briefly, dissection involved lateral cuts at the anterior and posterior end of the larva that encompassed 70% of larval circumference. These were followed by a longitudinal cut through the dorsal surface of the animal that connected the two lateral cuts. The intestines, other internal tissues, and neurons were then removed, and the flaps of tissue composed of epidermis and muscle were pinned down and fixed. For fixation, larvae were incubated in a solution of 10% formalin in PBS for 20 min.

#### Immunostaining

Staining of embryos and larvae was identical. Antibodies were used at the following dilutions: rabbit anti-dsRed (1:400, Clontech 632496), rat anti-tropomyosin (1:200, Abcam ab50567), mouse anti-GFP (1:50, Developmental Studies Hybridoma Bank GFP-G1), mouse anti-integrin βPS (1:50, Developmental Studies Hybridoma Bank CF.6G11), mouse anti-Fascin (1:25, Developmental Studies Hybridoma Bank sn 7C, generous gift from Tina Tootle), and mouse anti-αTubulin (1:200, Sigma-Aldrich T6199). Conjugated fluorescent secondary antibodies used were Alexa Fluor 555 donkey-anti-rabbit (1:200), Alexa Fluor 488 donkey-anti-rat (1:200), and Alexa Fluor 647 donkey-anti-mouse (1:200) (all Life Technologies) and Alexa Fluor 488 donkey-anti-mouse (1:200, Life Technologies). Furthermore, Acti-stain 555 phalloidin (1:400, Cytoskeleton PHDH1-A) and Hoechst 33342 (1 μg/ml) were used on larvae. Embryos and larvae were mounted in ProLong Gold (Life Technologies, P36930).

#### Microscopy

All microscopy was performed on a Zeiss LSM700 with an oil-immersion × 40 APOCHROMAT, 1.4 NA objective. All images of embryos, and the image of fascin localization in Fig. 2, were acquired with a 1.0× optical zoom. All other larval images were acquired with a 0.5× optical zoom. Image tiling was necessary to acquire images of the full larval muscles and was completed using the tiling function in the ZEN software that controls the microscope.

#### Statistics

All statistics were performed using GraphPad Prism. All data sets were compared to appropriate controls by a Student’s *t* test. *p < 0.05; **p < 0.01; ***p < 0.001; ****p < 0.0001.

### Image analysis

#### Analysis of nuclear position in larvae

Although the field has traditionally measured the distance between nuclei [18, 20, 21], this measurement does not account for changes in muscle size and nuclear number. We have therefore modified this measurement to determine how evenly nuclei are spaced within a muscle [22]. First, the area and length of the muscle was measured. Next, the position and number of nuclei is calculated by using the multipoint tool in ImageJ to place a point in the center of each nucleus. The position of each nucleus is used to calculate the actual internuclear distance. The maximal internuclear distance is determined by taking the square root of the muscle area divided by the nuclear number. This value represents the distance between nuclei, if internuclear distance was fully maximized. The ratio between the actual internuclear distance and the maximal internuclear distance ratio was then used to determine how evenly nuclei were distributed. This method normalizes the internuclear distance to both nuclear count and muscle area which leads to a more representative means of comparison between muscles, larvae, and genotypes. All viable (not torn) ventral longitudinal (VL3) muscles were measured from each larva. At least four larvae from one experiment were measured for each genotype. Statistical analysis was performed with Prism 4.0 (GraphPad). Student’s *t* test was used to assess the statistical significance of differences in measurements between experimental genotypes and controls.

#### Analysis of nuclear position in embryos

The position of nuclei was measured in stage 16 embryos. This is the latest stage before cuticle development blocks the ability to perform immunofluorescence microscopy. Embryos were staged based primarily on gut morphology as previously described [21]. At stage 16, the nuclei are reliably positioned adjacent to the muscle ends, and disruptions in this positioning can be easily determined as previously described [21, 23, 24]. Images, acquired as described above, were processed as maximum intensity projections of confocal z-stacks using ImageJ. The position of the nuclei was determined by using the line function in ImageJ to measure the distance between either the dorsal end of the muscle and the nearest nucleus or the ventral end of the muscle and the nearest nucleus. All four LT muscles were measured in four hemisegments from each embryo. At least 20 embryos from at least two independent experiments were measured for each genotype with each data point representing the average for all muscles measured within a single embryo.

#### Analysis of nuclear count in embryos

The number of nuclei was counted using apRed fluorescence in the 4 lateral transverse (LT) muscles of stage 17 embryos, once nuclei had separated from their clusters and into easily distinguished individual nuclei. Only hemisegments in which there were four lines of nuclei, corresponding to 4 LT muscles, were counted. Data points indicate the number of apRed positive nuclei within a single hemisegment. A total of at least 49 hemisegments from at least 19 individual embryos were counted.

#### Analysis of nuclear count in larvae

The number of nuclei in the VL3 muscles were counted using Hoechst staining. Data points indicate the number of nuclei within an individual muscle. Careful analysis of z-stacks was used to ensure that nuclei were in the muscle that they were attributed to.

#### Analysis of muscle attachment in embryos

Integrin accumulation at the myotendinous junction of stage 16 embryos were measured in dorsal muscle 2 (DO2). Z-stack projection images that extended through the entire MTJ were used for these data. Integrin fluorescence intensity line scans were measured within a 10 ×10 μm box at the segment border using plot profile function in ImageJ. The width of the fluorescence peak composed of fluorescence intensities greater than 25% of the maximum intensity (75% fluorescence intensity peak) was measured. Each data points indicate the width of the 75% fluorescence intensity peak for a single myotendinous junction. A total of at least 50 MTJs from 20 different embryos were analyzed.

#### Analysis of muscle size in larvae

The area of the VL3 muscles were measured using the polygon selection tool in ImageJ as previously described [22]. Data points indicate the size of an individual muscle.

### Analysis of general muscle architecture

Qualitative muscle phenotype analysis was completed on embryos of each genotype. All analysis was based on the immunofluorescence staining pattern of Tropomyosin in stage 16 embryos. The frequency of the following phenotypes were scored: the number of free myoblasts in an embryo that indicated a defect in myoblast fusion (small, unfused circles stained by anti-tropomyosin) and the number of muscles in each hemisegment (> 4 defined as extra muscles, < 4 defined as missing muscles) indicating gross abnormalities in the specification of muscle tissue. For analysis of the unfused myoblasts, embryos were grouped into bins with a width of 5 and the first bin centered on zero.

## Results

### Fascin is necessary for muscle function

**Fig. 1:**
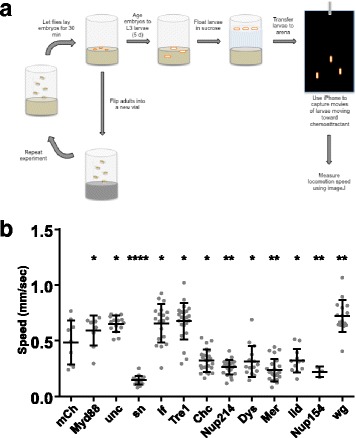
An RNAi screen for larval locomotion identifies fascin as a regulator of muscle function. **a** Cartoon illustrating the locomotion assay that was used to identify RNAi constructs that when expressed specifically in muscle, altered muscle function. **b** Graph indicating the speed of larval locomotion toward a chemoattractant when indicated genes were depleted by expression of RNAi specifically in muscle. All data were compared to their control by Student’s *t* test. *p < 0.05; **p < 0.01; ****p < 0.0001

Animal movement provides a simple assay for muscle function. Although adult locomotion has been used to perform a full-genome, RNAi-based screen for regulators of muscle function in *Drosophila* adults [17], similar screens have not been completed using *Drosophila* larvae. The greatest advantage to evaluating muscle function in larvae rather than adults is that pupation, and the high probability of lethality during pupation, is bypassed. We have therefore modified published larval locomotion assays [18, 19] to identify regulators of muscle function. To ensure that the identified genes had a muscle-autonomous effect on muscle function, we used the *GAL4/UAS* system [25] to disrupt gene function in a muscle specific manner. Specifically, we used *DMef2-GAL4* to drive the expression of a small library of UAS-RNAi constructs. We measured movement of larvae toward a chemo-attractant as previously described [19], with modifications to increase the throughput of the assay. First, we skipped the selection of stage 17 embryos, which previously ensured that the ages of the evaluated larvae were similar. We replaced this step, which previously took ~ 60 min per genotype, per experiment, with a timed-lay. Briefly, virgins that expressed *DMef2-GAL4* and males that carried the UAS-RNAi were mixed together in a vial for 1 h. The adults were then moved to another vial. The first vial, which contained all of the embryos that were laid during the 1-h period, was then used for the experiment to ensure that all larvae used in an experiment were of similar age. These vials were aged for 5 days until the animals were third-instar larvae (L3). Second, we measured the movement of many larvae simultaneously rather than measuring locomotion for an individual larva. The movement of larvae was then tracked using ImageJ (Fig. 1a). Measuring larval locomotion of many animals simultaneously provided two benefits. First, it increased the speed of the assay from ~ 60 min/genotype to ~ 10 min/genotype. Coincident with the increased speed of the assay, there was less variability in the age of larvae that were tracked in an experiment, thus increasing the precision of the data.

For the proof-of-concept screen, we expressed RNAi against 82 genes (Additional file 1: Table S1). The selected genes included those expected to impact muscle function (e.g., *Dystrophin*, *Msp300*) and many for which we did not have a prediction. The speed of locomotion in larvae that expressed each RNAi was compared to control larvae in which *DMef2-GAL4* drove the expression of mCherry RNAi. The disruption of 12/82 genes significantly altered larval locomotion compared to control larvae, indicating that these 12 genes regulate muscle function. Of these 12 genes, disruption of 5 caused larvae to move faster compared to controls, and the disruption of 7 caused the larvae to move more slowly than controls (Fig. 1b). RNAi directed against the expression of *singed* (*sn*) which encodes for the actin-binding protein fascin [26, 27] caused the greatest decrease in larval locomotion. Therefore, we investigated the impact that fascin depletion had on muscle structure to identify the mechanism by which fascin regulates muscle function.

### Fascin is localized to the nucleus in *Drosophila* muscle

**Fig. 2:**
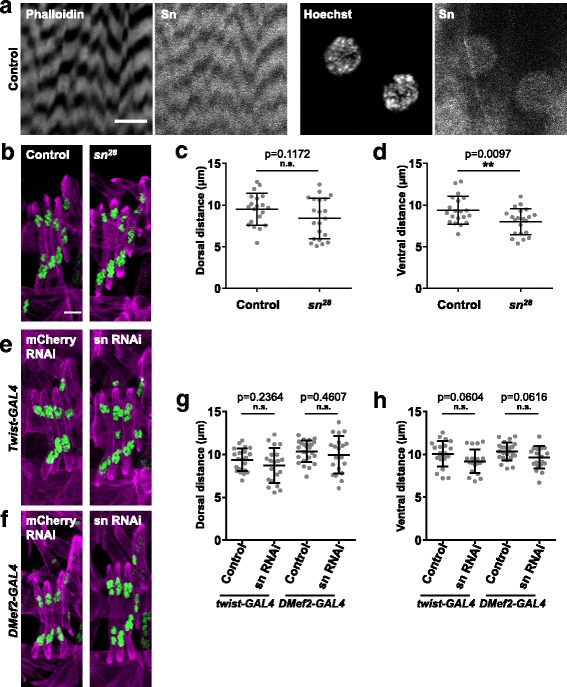
Fascin is localized to actin and the nucleus in muscle. **a** Images from different focal planes of dissected L3 larvae showing that fascin (sn) is colocalized with both Phalloidin (F-actin) (left two panels) and Hoechst (nuclei) (right two panels). **b** Immunofluorescence images from stage 16 embryos stained for Tropomyosin to identify the muscles (magenta) and the nuclei in the LT muscles (green) in control and *sn*^*28*^ mutant embryos. Scale bar, 10 μm. **c** Graph indicating the distance between the dorsal end of the muscle and the nearest nucleus in control and *sn*^*28*^ mutant embryos. **d** Graph indicating the distance between the ventral end of the muscle and the nearest nucleus in control and *sn*^*28*^ mutant embryos. **e**, **f** Immunofluorescence images from stage 16 embryos stained for Tropomyosin to indicate the muscles (magenta) and the nuclei within the LT muscles (green) in embryos where RNAi against either mCherry (control) or fascin (sn RNAi) was driven by *Twist-GAL4* (**e**) or *Dmef2-GAL4* (**f**). **g**, **h** Graphs indicating the average distance between the dorsal end of the muscle and the nearest nucleus (**g**) or the ventral end of the muscle and the nearest nucleus (**h**) in embryos of indicated genotypes. All data (**c**, **d**, **g**, **h**) were compared to their control by Student’s *t* test. **p < 0.01

As a first approach to determine how fascin regulates muscle function, we examined the localization of fascin in *Drosophila* larval muscles and found that fascin localized to the sarcomeres and to the nuclei. The nuclear localization is similar to the localization of fascin in nurse cells [28] (Fig. 2a).

An emergent regulator of muscle function is the position of the many myonuclei within a single cytoplasm [12, 13, 29]. Based on the localization of fascin to the nucleus, we hypothesized that fascin may regulate nuclear movement during muscle development. Consistent with this hypothesis, fascin interacts with the nuclear envelope protein Nesprin-2 and regulates nuclear movement in migrating fibroblasts [30] and is necessary for the positioning of nuclei in developing *Drosophila* oocytes [28]. To determine whether nuclear position was affected in *Drosophila* muscle, we crossed apRed, a marker for the nuclei in the lateral transverse (LT) muscles [31] into the *sn*^*28*^ *Drosophila* and measured the position of nuclei as previously described [21]. In *sn*^*28*^ mutants, nuclei were closer to the ventral end of the muscle compared to controls (Fig. 2b–d). To determine whether the effect on nuclear position was muscle autonomous, we transiently expressed RNAi specifically in the mesoderm during the early stages of muscle development using *Twist-GAL4* or in a more sustained manner using *DMef2-GAL4* and measured the position of the nuclei. Muscle-specific depletion of fascin had no impact on nuclear position (Fig. 2e–h). Together, these data indicate that although nuclei are closer to the muscle end in fascin mutants, this is not regulated by fascin expressed in muscle during embryonic muscle development in *Drosophila*.

### Fascin regulates myoblast fusion

**Fig. 3:**
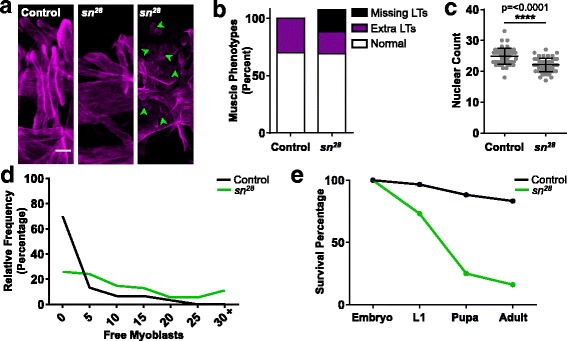
Fascin is necessary for myoblast fusion. **a** Immunofluorescence images of stage 16 embryos stained for Tropomyosin to indicate the muscles (magenta). Images are focused on the lateral transverse (LT) muscles in stage 16 embryos. Green arrowheads indicate unfused myoblasts. Scale bar, 10 μm. **b** Graph indicating the percentage of embryos that had at least one hemisegment with > 4 LT muscles (Extra LTs) or < 4 LT muscles (missing LTs). Values that exceed 100% indicate that some embryos had one hemisegment with > 4 LT muscles (extra muscles) and another hemisegment with < 4 LT muscles (missing muscles). **c** Graph indicating the number of apRed positive nuclei per hemisegment. Student’s *t* test was used for comparison to controls. ****p < 0.0001. **d** Graph indicating how frequently different numbers of unfused myoblasts are seen in control (black) and *sn*^*28*^ mutant embryos (green). **e** Graph indicating the viability of the *sn*^*28*^ mutant animals (green) compared to controls (black)

Because the loss of fascin had a limited effect on nuclear position in *Drosophila* embryonic muscles, we looked at the general muscle pattern in the *sn*^*28*^ mutant embryos. At embryonic stage 16, there are 30 well-characterized muscles per hemisegment in the *Drosophila* embryo [32]. We noted a number of differences between *sn*^*28*^ mutant embryos and controls (Fig. 3a). First, there was a reduction in the number of muscles. Although, various muscles were missing in individual hemisegments, we focused on the LT muscles because they are near the embryo surface and are the only muscles that are perfectly aligned on the dorsal-ventral axis of the embryo. These features make the LTs easy to count, image, and analyze.

The typical hemisegment from a control embryo has four LT muscles. Seventy percent of control embryos had four LT muscles in every hemisegment, and 30% of controls had at least one hemisegment with greater than four LT muscles. In *sn*^*28*^ mutants, 70% of embryos had four LT muscles in every hemisegment and 19% of embryos had at least one hemisegment with greater than four LT muscles. Additionally, 19% of *sn*^*28*^ embryos had at least one hemisegment with fewer than four LT muscles (Fig. 3b), and were therefore missing LT muscles. Eight percent of embryos had at least one hemisegment with an extra muscle and one hemisegment with a missing muscle and were thus counted in both categories, pushing the total embryos over 100%. Because the absence of muscles can indicate a defect in myoblast fusion, we counted the number of nuclei that were incorporated into the LT muscles per hemisegment. This number was reduced from a mean of 26 in the controls to a mean of 22 in the *sn*^*28*^ mutants (Fig. 3c). Consistent with this, there was an increase in unfused myoblasts. In controls, the median number of free myoblasts per embryo was 1, and that number increased to 7.5 in *sn*^*28*^ mutants (Fig. 3d). Additionally, 70% of control embryos had two or fewer identifiable unfused myoblasts whereas 75% *sn*^*28*^ mutant embryos had at least three unfused myoblast and 50% of *sn*^*28*^ mutant embryos eight or more unfused myoblasts. Based on the missing muscles and the abundance of unfused myoblasts in mutant embryos, we tested the viability of the *sn*^*28*^ mutants and found that there was significant lethality during both the embryonic and larval stages (Fig. 3e). These data indicate that fascin regulates myoblast fusion.

To determine whether these phenotypes were muscle autonomous, we used the *GAL4/UAS* system to deplete fascin specifically from the developing mesoderm and muscle of the *Drosophila* embryo. The expression of a UAS-sn RNAi (fascin RNAi) was driven with each of three GAL4 drivers. *Twist-GAL4* was used to drive RNAi expression in the early mesoderm, *DMef2-Gal4* was used to drive RNAi expression slightly later in muscle with sustained expression throughout development, and *MHC-GAL4* was used to drive RNAi expression beginning at the final stage of embryonic development and continuing throughout development. We then examined the general muscle structure in stage 16 embryos as we had done for *sn*^*28*^ mutant embryos. There were no defects in muscle morphology when *MHC-GAL4* was used to drive RNAi expression suggesting that fascin must be depleted early during development to have significant impact (Additional file 2: Figure S1).

**Fig. 4:**
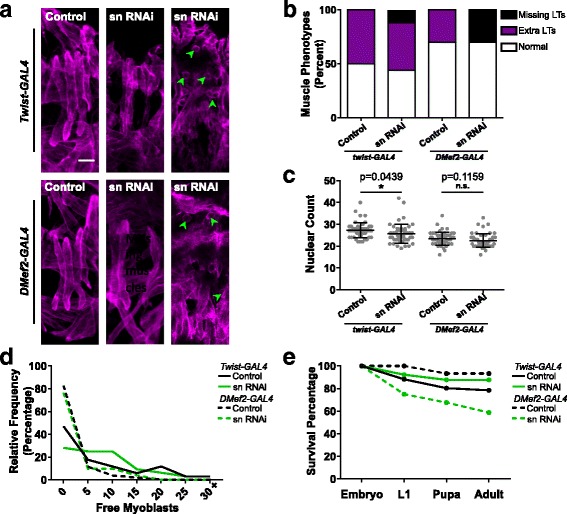
Fascin has muscle autonomous effects on myoblast fusion. **a** Stage 16 embryos stained for tropomyosin to identify the muscles (magenta). Embryos expressed RNAi against fascin under the control of *Twist-GAL4* (top) or *Dmef2-GAL4* (bottom). Green arrowheads indicate unfused myoblasts. Scale bar, 10 μm. **b** Graph indicating the percentage of embryos of indicated genotypes that have at least one hemisegment with either > 4 LT (extra LTs) muscles or < 4 LT muscles (missing LTs). **c** Graph indicating the number of apRed positive nuclei incorporated into LT muscles per hemisegment in indicated genotypes. Student’s *t* test was used for comparison to controls. *p < 0.05. **d** Graph indicating how frequently different numbers of unfused myoblasts were seen in indicated genotypes. **e** Graph indicating the viability of animals with indicated genotypes

Early mesodermal expression of the RNAi under the control of *Twist-GAL4* and expression of RNAi in muscle under the control of *DMef2-GAL4* both increased the percentage of embryos that were missing LT muscles, but *DMef2-GAL4*-mediated expression resulted in a higher frequency of embryos with missing muscles (Fig. 4a, b). Conversely, only *Twist-GAL4*-mediated expression of RNAi against fascin caused a decrease in the number of nuclei that were incorporated into the LT muscles (Fig. 4c). This suggested that early expression of fascin RNAi was necessary to inhibit myoblast fusion. Consistent with this, *Twist-GAL4*-mediated RNAi expression increased both the percentage of embryos with unfused myoblasts and the number of unfused myoblasts per embryos. *DMef2-GAL4*-mediated expression of fascin RNAi affected neither measure of fusion (Fig. 4d). However, *DMef2-GAL4*-mediated expression of RNAi had a greater effect on viability (Fig. 4e) suggesting that the absence of muscles was more detrimental to animal viability.

### Fascin-dependent fusion effects are evident in larvae

**Fig. 5:**
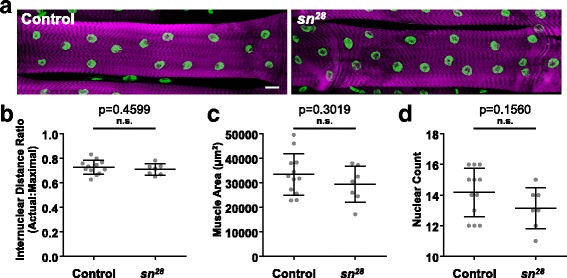
Genetic disruption of fascin did not affect larval muscle structure. **a** Immunofluorescence images of the third ventral longitudinal muscle (VL3) in L3 larvae of indicated genotypes. Sarcomeres (magenta) were stained with Phalloidin to identify the muscle and Hoechst (green) was used to identify the nuclei. Scale bar, 25 μm. **b** Graph indicating the actual internuclear distance divided by the maximal internuclear distance in indicated genotypes. **c** Graph indicating the area of the muscles as a proxy for muscle size in the indicated genotypes. **d** Graph indicating the number of nuclei in indicated genotypes. All data (**b**–**c**) were compared to their control by Student’s *t* test. All differences were statistically insignificant

To determine the effects of fascin-depletion later in development, larvae were dissected and stained with Phalloidin to identify the muscles and Hoechst to identify the nuclei (Fig. 5). We examined the third ventral longitudinal muscle (VL3) because after dissection this muscle is on the surface and therefore easily imaged. The distribution of myonuclei was similar in the controls and *sn*^*28*^ larvae (Fig. 5a, b). The size of the muscles (Fig. 5a, c), and the number of nuclei in each muscle (Fig. 5a, d) were both reduced in the *sn*^*28*^ larvae compared to the controls, but the reductions were statistically insignificant. We hypothesized that the lack of phenotype may be based on selection of the healthiest animals because they are the animals that survived until the L3 stage. As such, we examined animals that expressed RNAi specifically in the muscle, which are more viable (compare Fig. 4e to Fig. 3e).

**Fig. 6:**
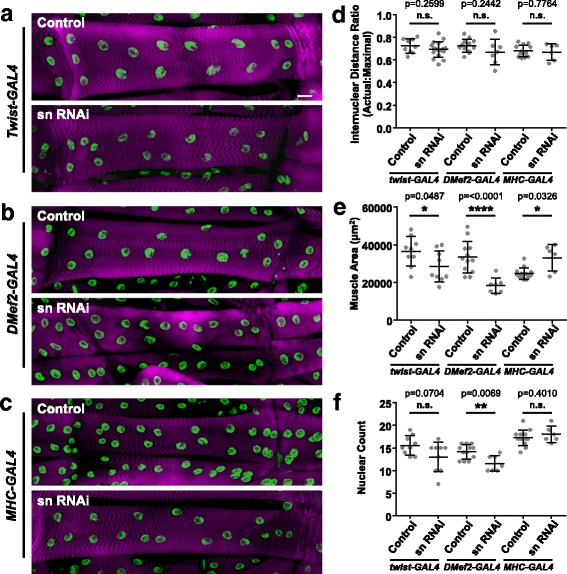
Muscle-specific depletion of fascin results in smaller muscles with fewer nuclei. **a**–**c** Immunofluorescence images of the VL3 muscle in L3 larvae that expressed RNAi against either mCherry (control) or fascin (sn RNAi) under the control of *Twist-GAL4* (**a**), *DMef2-GAL4* (**b**), or *MHC-GAL4* (**c**). Sarcomeres were identified by Phalloidin (magenta), and nuclei were identified by Hoechst (green). Scale bar, 25 μm. **d** Graph indicating the actual internuclear distance divided by the maximal internuclear distance in indicated genotypes. **e** Graph indicating the area of the muscles in larvae of indicated genotypes. **f** Graph indicating the number of nuclei per muscle in larvae of indicated genotypes. All data (**d**–**e**) were compared to their control by Student’s *t* test. *p < 0.05; **p < 0.01; ****p < 0.0001

We expressed RNAi against fascin under the control of *Twist-GAL4* (early, transient expression), *DMef2-Gal4* (slightly later, sustained expression), or *MHC-Gal4* (late, sustained expression). The distribution of nuclei was the same in each genotype (Fig. 6a–d). Muscle size was decreased when either *Twist-GAL4* or *DMef2-GAL4* was used to express fascin RNAi (Fig. 6e) suggesting that early fascin-dependent processes contribute to fascin-dependent muscle growth. Finally, the number of nuclei in VL3 muscles was reduced by *DMef2-GAL4*-mediated fascin depletion. *Twist-GAL4*-mediated depletion did reduce the number of nuclei per muscle, but insignificantly so. *MHC-GAL4*-mediated depletion had no impact on the number of nuclei per muscle (Fig. 6a–c, f). Thus, the defects in fusion are not transient, but are evident throughout larval development.

### Fascin regulates muscle attachment

**Fig. 7:**
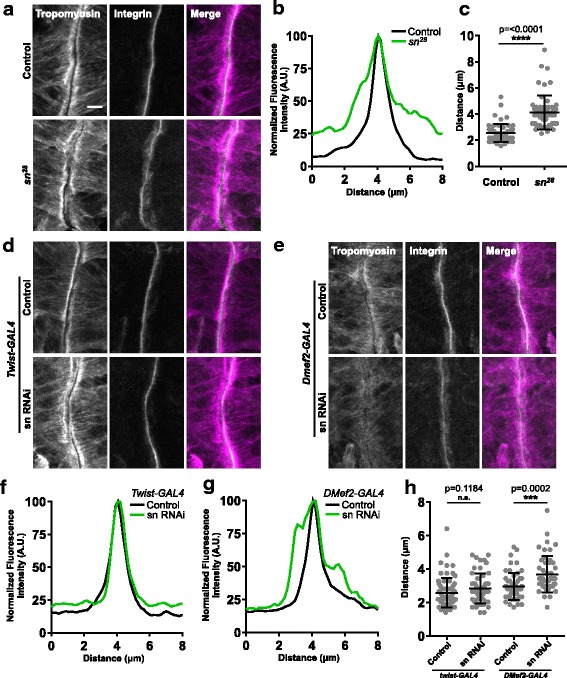
Fascin is necessary for proper myotendinous junction organization **a** Immunofluorescence images of the MTJ of muscle DO2 stained for tropomyosin and βPS-integrin in control (top) and *sn*^*28*^ mutant embryos (bottom). Scale bar, 10 μm. **b** Representative intensity profile of the βPS-integrin signal in control (black) and *sn*^*28*^ mutant embryos (green). **c** Graph indicating the width of the βPS-integrin signal defined by the points at which the signal is 25% of the maximum. **d**, **e** Immunofluorescence images of the MTJ of muscle DO2 stained for tropomyosin and βPS-integrin in animals in which twist-GAL4 was used (**d**) or DMef2-GAL4 was used (**e**) to express RNAi against either mCherry (control) or fascin (sn RNAi). **f**, **g** Representative intensity profiles of the βPS-integrin signal in indicated genotypes. **h** Graph indicating the width of the βPS-integrin signal in indicated genotypes as defined by the points at which the signal is 25% of the maximum. All data (**c**, **h**) were compared to their control by Student’s *t* test. **p < 0.01; ****p < 0.0001

*DMef2-Gal4*-mediated expression of sn RNAi did not reduce the number of nuclei incorporated into embryonic LT muscles (Fig. 4c), but did reduce the total number of muscles in the embryo (Fig. 4b). This could be explained by an effect on the attachments between the muscle and the tendon cell at the myotendinous junction (MTJ). To determine whether fascin affected MTJ integrity, we immunostained embryos for tropomyosin to identify the muscles and βPS-integrin to identify the MTJ (Fig. 7). We measured the width of the βPS-integrin signal at the MTJ of dorsal muscle 2 (DO2). Compared to controls, the signal was wider in *sn*^*28*^ mutants (Fig. 7a–c). Similarly, *DMef2-GAL4*-mediated expression of fascin RNAi, but not Twist-GAL4-mediated expression of fascin RNAi also increased the width of the βPS-integrin signal (Fig. 7d–h). These data suggest that sustained fascin function is necessary for proper MTJ organization.

## Discussion

One of the many strengths of *Drosophila* as a model system is its utility as a tool to identify novel regulators of specific biological functions. This ability utilizes the immense genetic tools that are available and requires simple and fast assays to screen many mutants and/or RNAi lines. In this work, we adapted a published larval-tracking assay [19] to perform a proof-of-concept screen for muscle function. We identified 12 genes that regulate muscle function, either positively or negatively. We continued these experiments by examining the mechanism by which *singed*, *Drosophila* fascin, regulated muscle function because fascin-depletion had the strongest effect on muscle function.

We used a combination of mutant alleles and tissue-specific expression of RNAi against fascin to demonstrate that fascin regulates both myoblast fusion and the structure of the MTJ. Fascin is well-described as a protein that can bundle F-actin filaments and increase their strength, and the strength of actin-based cellular protrusions [33]. Furthermore, by this mechanism, fascin contributes to cellular invasions associated with cancer metastasis [34, 35]. Myoblast fusion requires a similar organization of protrusive F-actin structures that invade the growing myotube. The most surprising aspect of the myoblast fusion data is the relatively minor effect that fascin has compared to other genes necessary for myoblast fusion [31, 36–38]. The reason for this is not clear. One possibility is that maternal loading provides sufficient fascin to facilitate the initial rounds of fusion. Alternatively, perhaps the final fusion events require greater protrusive force and only then does the function of fascin become critical.

The contribution of fascin to MTJ structure is consistent with previously published data. Fascin contributes to filopodia formation [35] and MTJ development is dependent on filopodia-like extensions. Furthermore, although the MTJ forms as a smooth attachment during pupation [16], the MTJ in the embryo is dynamic [39]. Thus, perhaps fascin is continually necessary for the turnover and the integrity of the MTJ.

Perhaps the most intriguing feature of these data is the temporal separation of fascin-dependent myoblast fusion and fascin-dependent MTJ stability. This conclusion is based on our finding that the time and duration of fascin depletion determines the phenotype that will emerge. Transient depletion of fascin during early stages of muscle development disrupted myoblast fusion but not MTJ structure. Conversely, later, and sustained depletion of fascin affected MTJ structure, but not myoblast fusion. These data are important because they demonstrate that although both fusion and MTJ structure require fascin function, they are not codependent features of muscle development.

It is not clear whether either function is more critical than the other. Certainly, sustained depletion of fascin, which disrupts MTJ integrity has a greater effect on animal survival than does the transient depletion that disrupts fusion. However, this conclusion is limited because the impact that a small reduction in nuclear number has on muscle organization is not clear. Reduced nuclear numbers do correlate with reduced muscle size [40, 41] and therefore likely cause reduced muscle function. Data in embryos indicated that *DMef2*-*GAL4*-mediated expression of fascin RNAi only affected MTJs and would therefore allow us to isolate the impact of the MTJ versus the impact of myoblast fusion. However, we see that in larvae, there is a reduction in the number of nuclei per muscle. Because there is no repair of embryonic and larval muscles in *Drosophila*, we suspect that this reduction is based in muscle damage that may be linked to improper attachments and poor mechanical stability. However, further work is necessary to understand the mechanism by which nuclei are lost so that the impact of individual fascin-dependent functions can be determined.

## Conclusions

In total, we have used a larval locomotion assay to identify novel regulators of muscle function. Furthermore, we have described that the strongest hit from the screen, fascin, contributes to muscle function by regulating myoblast fusion and MTJ structure. Both of these functions are consistent with known biochemical abilities for fascin and suggest that fascin has multiple essential functions for muscle development that are separated in time. More generally, this work outlines a new strategy for the identification of genes and pathways that can be manipulated to either increase or decrease muscle function.

## Additional files

Additional file 1: **Table S1.** All of the data acquired during the limited RNAi-based screen for regulators of muscle function. (PDF 139 kb)

Additional file 2: **Figure S1.** Expression of RNAi against fascin late in embryonic development does not affect muscle development a Immunofluorescence images showing the muscle pattern in animals expressing mCherry RNAi (control) and animals expressing fascin RNAi (sn RNAi) under the control of the *MHC-GAL4* driver. b Graph comparing the frequency of embryos with extra muscles in each genotype. No embryos with missing muscles were observed in either genotype. c Graph comparing the frequency at which embryos were found to have unfused myoblasts in each genotype. (PDF 360 kb)
