# Supplementary material for: An RNAi based screen in Drosophila larvae identifies fascin as a regulator of myoblast fusion and myotendinous junction structure
Source: Skelet Muscle. 2018 Apr 6;8:12. doi: 10.1186/s13395-018-0159-9 (PMC5889537; doi:10.1186/s13395-018-0159-9)
Supplement: Supplementary file 1 — Table S1. All of the data acquired during the limited RNAi-based screen for regulators of muscle function. (PDF 139 kb) [file 13395_2018_159_MOESM1_ESM.pdf]

Drivers	TRiP	Data Count	Minute 3 Speed	Minute 3 St.Dev	t-test
DMef2Gal4	mCh	9	0.484318519	0.197186589	
DMef2Gal4	Alk	11	0.330749311	0.070968181	0.05135355
DMef2Gal4	eyg	10	0.449525	0.093293016	0.63848877
DMef2Gal4	not	18	0.389675	0.223676971	0.27603277
DMef2Gal4	Nup75	16	0.606995833	0.143446012	0.12554894
DMef2Gal4	slou	10	0.467798333	0.145316198	0.83963965
DMef2Gal4	tin	19	0.486021053	0.151405086	0.98208623
DMef2Gal4	jar	18	0.444092593	0.126288438	0.58797649
DMef2Gal4	N	10	0.603808333	0.126876691	0.14400199
DMef2Gal4	nwk	14	0.451880952	0.134326988	0.67215023
DMef2Gal4	Tollo	19	0.583319298	0.135580375	0.19896387
DMef2Gal4	Shot	14	0.462590476	0.123988125	0.77285928
DMef2Gal4	bnl	1	0.629933333		
DMef2Gal4	Myd88	9	0.780703623	0.237116199	0.00209739
DMef2Gal4	shu	23	0.572036232	0.102891172	0.23399634
DMef2Gal4	unc	15	0.652436667	0.07272363	0.0353297
DMef2Gal4	Cac	23	0.557966667	0.120771273	0.31903586
DMef2Gal4	wupA	22	0.708602273	0.128675018	0.00935297
DMef2Gal4	Nup98-96	21	0.650319841	0.220300923	0.05744977
DMef2Gal4	Tre1	23	0.675723913	0.165144242	0.02329149
DMef2Gal4	Rac1	18	0.52365	0.113874947	0.59092904
DMef2Gal4	Vang	20	0.531365833	0.132941847	0.52720872
DMef2Gal4	wg	16	0.722467708	0.143763863	0.0073269
DMef2Gal4	sn	21	0.146266667	0.036393413	0.00084398
DMef2Gal4	if	19	0.656307895	0.171997361	0.04156853
DMef2Gal4	Him	20	0.4544925	0.138091105	0.68871839
DMef2Gal4	Apc	23	0.561425362	0.134842061	0.30373954
DMef2Gal4	Egfr	15	0.500688889	0.200547194	0.84718063
DMef2Gal4	mael	23	0.356563043	0.110694421	0.09644317
DMef2Gal4	smo	22	0.500797727	0.080826131	0.81374364
DMef2Gal4	vvl	23	0.429733333	0.123181669	0.45622448
DMef2Gal4	Insc	4	0.463504167	0.141333184	0.83454401
DMef2Gal4	Chc	23	0.3222	0.100393582	0.041446468
DMef2Gal4	Nup214	23	0.262912319	0.066661251	0.009695968
DMef2Gal4	mus	23	0.428624638	0.118652987	0.44556685
DMef2Gal4	mbl	23	0.429234783	0.120445489	0.45113419
DMef2Gal4	Rbf	23	0.511402899	0.131314596	0.71097455
DMef2Gal4	Dys	15	0.313902222	0.140440293	0.040962822
DMef2Gal4	Akt1	14	0.479138095	0.084186984	0.94204412
DMef2Gal4	lac	20	0.369674167	0.121764046	0.1357798

DMef2Gal4	Max	10	0.398453333	0.132995847	0.28995135
DMef2Gal4	tsr	21	0.60314127	0.192961358	0.14888353
DMef2Gal4	lmp	6	0.418377778	0.115305357	0.42953098
DMef2Gal4	raps	6	0.357316667	0.078412133	0.1096662
DMef2Gal4	Mer	23	0.237402899	0.101337394	0.005290206
DMef2Gal4	phl	22	0.334489394	0.158406138	0.06456794
DMef2Gal4	foxoB25997	10	0.409451667	0.11495148	0.3376516
DMef2Gal4	lid	12	0.321336111	0.107364126	0.045408071
DMef2Gal4	hh	13	0.546273077	0.125833648	0.42076512
DMef2Gal4	Cg25C	22	0.534558333	0.176463681	0.5182251
DMef2Gal4	SoxN	1	0.246083333		
DMef2Gal4	Nup154	3	0.221783333	0.046176666	0.004250752
DMef2Gal4	lms	23	0.508844928	0.107727273	0.73139874
DMef2Gal4	Nup133	21	0.491593651	0.153817524	0.92305429
DMef2Gal4	E2F	8	0.595639583	0.172774985	0.23380661
DMef2Gal4	Mad	23	0.493397101	0.133237569	0.90106191
DMef2Gal4	Scaf	23	0.55526087	0.151029677	0.34979557
DMef2Gal4	ea	22	0.512959091	0.153990532	0.70339249
DMef2Gal4	beat-la	22	0.430246212	0.185709234	0.49244434
DMef2Gal4	nmo	23	0.52343913	0.127320692	0.59235881
DMef2Gal4	p53	23	0.603881159	0.111936406	0.11700875
DMef2Gal4	mtm	23	0.630017391	0.151591664	0.06914109
DMef2Gal4	Dsor1	23	0.539943478	0.126565183	0.44931508
DMef2Gal4	Tsc1	23	0.636095652	0.159494709	0.06111673
DMef2Gal4	Myo3DF	19	0.592053509	0.12566915	0.16102503
DMef2Gal4	Nos	9	0.545481481	0.11753778	0.4384215
DMef2Gal4	foxoB23	23	0.579510145	0.105786192	0.20017617
DMef2Gal4	rhea	15	0.626348889	0.130691324	0.07805901
DMef2Gal4	nau	15	0.592858889	0.111738888	0.15834268
DMef2Gal4	Nup50	21	0.425521429	0.13026535	0.42887561
DMef2Gal4	betaTub56D	20	0.524993333	0.139955583	0.5868077
DMef2Gal4	Ote	23	0.44444058	0.151559329	0.59463405
DMef2Gal4	Koi	11	0.428819697	0.15752762	0.50402665
DMef2Gal4	Klar	23	0.581669565	0.191922776	0.22605245
DMef2Gal4	LamC	10	0.562736667	0.117337754	0.31812136
DMef2Gal4	Act5C	Lethal			
DMef2Gal4	Vrp1	Lethal			
DMef2Gal4	Twi	Lethal			
DMef2Gal4	Imp	Lethal			
DMef2Gal4	Nup153	Lethal			
DMef2Gal4	Nup160	Lethal			

DMef2Gal4	Wit	Lethal
DMef2Gal4	Wts	Lethal
DMef2Gal4	Vkg	Lethal
