# Supplementary figures and images for: An RNAi based screen in Drosophila larvae identifies fascin as a regulator of myoblast fusion and myotendinous junction structure
Source: Skelet Muscle. 2018 Apr 6;8:12. doi: 10.1186/s13395-018-0159-9 (PMC5889537; doi:10.1186/s13395-018-0159-9)

A

MHC-GAL4

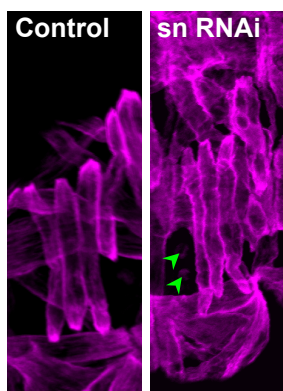

B

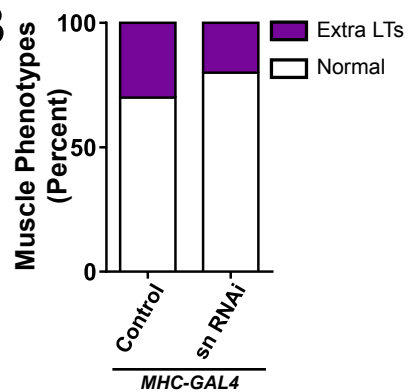

C

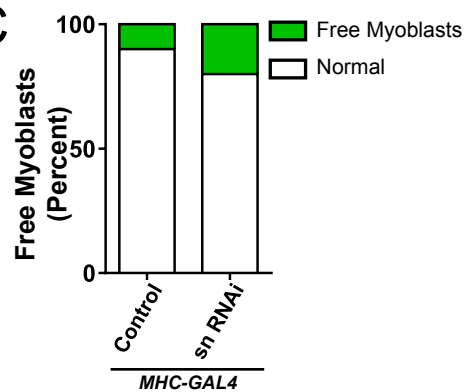

Camuglia et al., Supp Figure

Supplement: Supplementary file 2 — Figure S1. Expression of RNAi against fascin late in embryonic development does not affect muscle development a Immunofluorescence images showing the muscle pattern in animals expressing mCherry RNAi (control) and animals expressing fascin RNAi (sn RNAi) under the control of the MHC-GAL4 driver. b Graph comparing the frequency of embryos with extra muscles in each genotype. No embryos with missing muscles were observed in either genotype. c Graph comparing the frequency at which embryos were found to have unfused myoblasts in each genotype. (PDF 360 kb) [file 13395_2018_159_MOESM2_ESM.pdf]
